# Bladder health and the urogenital microbiome in community-dwelling adult females

**DOI:** 10.1128/msystems.00558-25

**Published:** 2025-10-16

**Authors:** L. Brubaker, D. McDonald, S. Putnam, C. Brennan, C. S. Fok, C. E. Lewis, J. L. Lowder, M. G. Mueller, L. M. Rickey, E. R. Mueller, S. J. Song, K. Rudser, R. Knight, E. S. Lukacz, for the PLUS Consortium

**Affiliations:** 1 Department of Obstetrics, Gynecology, and Reproductive Sciences, UC San Diego School of Medicine, University of California San Diego 197204, La Jolla, California, USA; 2 Department of Pediatrics, University of California San Diego 547075, La Jolla, California, USA; 3 School of Public Health, Division of Biostatistics and Health Data Science, University of Minnesota 43353, Minneapolis, Minnesota, USA; 4 Division of Biological Sciences, University of California San Diego 8784, La Jolla, California, USA; 5 Department of Urology, University of Minnesota 5635, Minneapolis, Minnesota, USA; 6 Department of Epidemiology, School of Public Health, University of Alabama at Birmingham 48653, Birmingham, Alabama, USA; 7 Department of Obstetrics and Gynecology, Washington University in St. Louis School of Medicine 12275 , St. Louis, Missouri, USA; 8 Department of Obstetrics and Gynecology, University of Chicago, Pritzker School of Medicine 12246, Chicago, Illinois, USA; 9 Department of Urology, Yale University School of Medicine, New Haven, Connecticut, USA; 10 Department of Obstetrics and Gynecology, Loyola University Chicago Stritch School of Medicine, Chicago, Illinois, USA; 11 Center for Microbiome Innovation, Jacobs School of Engineering, University of California San Diego 8784, La Jolla, California, USA; 12 Department of Bioengineering, University of California San Diego 8784, La Jolla, California, USA; 13 Department of Computer Science and Engineering, University of California San Diego 8784, La Jolla, California, USA; 14 Halıcıoğlu Data Science Institute, University of California San Diego 8784, La Jolla, California, USA; Cleveland Clinic, Cleveland, Ohio, USA

**Keywords:** bladder health, urobiome, microbiome, urinary, female

## Abstract

Despite the established association with lower urinary tract symptoms, the relationship between a healthy bladder and the urogenital microbiome is unknown. This observational cohort study of 435 community-dwelling women examined bladder health and function using a validated instrument. Voided urine samples were self-collected, shipped to a repository, and underwent 16S rRNA amplicon sequencing. Two hundred seventy-four (56%) samples were retained following a limit-of-detection analysis for low biomass samples. A differential abundance analysis showed that *Lactobacillus,* the dominant genus, was associated with higher bladder function scores, but not global perception of bladder health, while *Prevotella* and *Anerococcus* were associated with lower scores. Associations between the female urogenital microbiome exist across the bladder health spectrum.

**IMPORTANCE:**

There is increasing awareness that human microbiomes impact health and modulate certain health conditions. Recently, investigators developed a validated assessment of bladder health in adult women. This advance facilitated evaluation of the urogenital microbiome, across the adult lifespan and across the spectrum of bladder health in a population-based, observational study.

## INTRODUCTION

Since the discovery and confirmation of the urinary microbiome, commonly called the urobiome, over a decade ago, several studies have demonstrated that urinary tract conditions, symptoms, and treatment responses are associated with the urobiome (1–3). Urobiome is an overarching term used to denote the microbial communities of the urinary tract. Consistent with current terminology, the term “urogenital urobiome” denotes voided urine samples that reflect the bladder and urethral urobiomes with contributions from the external genitalia.

However, existing foundational studies, including benchmarking and reporting recommendations, are limited by a focus on symptomatic and disease states without a larger context of the urobiome in bladder health states (4). Traditionally, studies have considered the absence of the condition of interest (e.g., urgency urinary incontinence vs no urinary incontinence) as an adequate proxy for a “healthy” bladder status when comparing the urobiome in diseased vs “control” populations. This decision was pragmatic, as there were no validated methods for determining a healthy bladder. However, there is a broad understanding that the absence of symptoms or disease does not equate with “health.”

In response to a need to understand what constitutes a “healthy bladder,” in 2015, the National Institute of Diabetes and Digestive and Kidney Diseases-supported Prevention of Lower Urinary Tract Symptoms (PLUS) Research Consortium convened a group of experts from across the country to define bladder health, develop instruments to measure it, and to identify factors associated with both health and disease (5, 6). The resulting Bladder Health Scales (BHS) and the Bladder Function Indices (BFI) were developed and validated for use in population-based research and designed to quantify bladder health and function on a continuous spectrum from poor (0) to optimal (100) health (7). The BHS also allows for adjustment of scores based on an individual’s adaptive behaviors such as toilet mapping and pad usage, which can lead to an overestimate of an individual’s perception of their own bladder health. The distribution of bladder health and function has been reported in a large population-based study of adult women across nine regions of the United States (8).

The factors of interest associated with this spectrum of bladder health included social, demographic, medical, and biological factors, including the urobiome. The PLUS Research Consortium recognized the challenges of urobiome research at the population level due to numerous factors including the low biomass environment, challenges with specimen collection, storage and shipping as well as laboratory techniques, analytic approaches, and scientific interpretation. As such, foundational studies were conducted that demonstrate the feasibility of self-collection, shipment, processing, and analysis among community-dwelling women (9, 10).

In this analysis, we examined the urogenital microbiome’s relationship to the following three measures of bladder health: (i) the global Bladder Health Scale, (ii) the global Bladder Health Scale with adjustment for behavioral adaptation (global Bladder Health ABA scale), and (iii) the total Bladder Function Index score, which averages six bladder function scores across urinary infections, frequency, sensation, continence, and comfort. We hypothesized that urogenital microbiome characteristics would differ between study participants with higher bladder health and function scores compared to those with lower bladder health and function. Adult, community-dwelling women participants contributed data for a large cohort study conducted by the PLUS Research Consortium (RISE FOR HEALTH study [RISE]) (8).

## RESULTS

### Participants’ characteristics and bladder health scores

**Fig 1:**
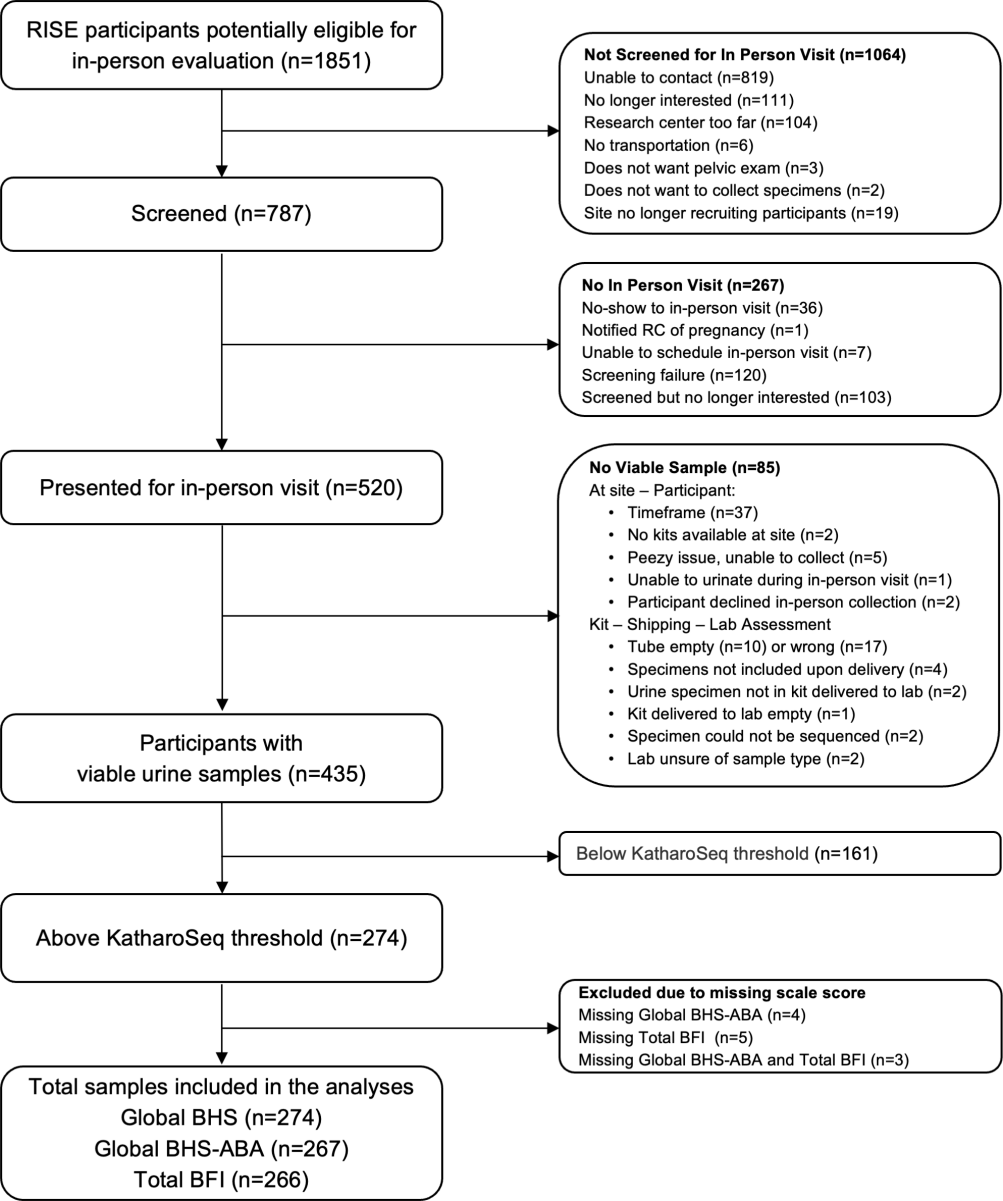
Flow chart depicting participants and self-collected urine sample for urobiome analysis.

Figure 1 depicts the flow of participant samples for this analysis. Of the 520 participants that presented for the in-person visit, 513 had a urine specimen tube sent to the lab by the research coordinator. Of those 513 urine specimen tubes, 78 were not viable for sequencing analysis. The 435 participants with viable urine samples for sequencing had a mean (SD) Global Bladder Health score of 66.2 (20.3), Global Bladder Health-ABA scale score of 53.1 (25.4), and a total Bladder Function Index score of 72.0 (17.0) (Table 1). Of these, 274 (63%) urine samples were above the pre-determined 80% KatharoSeq sequencing threshold and retained for downstream analysis. This proportion is in alignment with expectations for similarly collected, low-biomass urine samples. KatharoSeq is a method for determining the limit of detection within a set of samples using a positive control, resulting in a defined threshold for excluding samples under a certain sequencing depth. For this study, we used the 80% threshold, meaning that the exclusion depth was determined as that where 80% of the reads in the positive control could be mapped to the organism contained within the control. The participants with samples below the threshold tended to be older, and a larger percentage were white. As this comparison was not specified *a priori* and does not correspond to any hypothesis of interest, no statistical hypothesis testing was conducted.

**TABLE 1:** Descriptive summary of participants with a sequenced self-collected urine sample

	Above KatharoSeq threshold	Not above KatharoSeq threshold	Sequenced
*n*	274	161	435
Age group (yr; four levels) (%)			
18–25	36 (13.1)	12 (7.5)	48 (11.0)
26–44	85 (31.0)	39 (24.2)	124 (28.5)
45–64	95 (34.7)	73 (45.3)	168 (38.6)
65+	56 (20.4)	37 (23.0)	93 (21.4)
Missing	2 (0.7)	0 (0.0)	2 (0.5)
Race/Ethnicity (mutually exclusive categories) (%)			
Asian	14 (5.1)	12 (7.5)	26 (6.0)
Black	47 (17.2)	11 (6.8)	58 (13.3)
Hispanic	34 (12.4)	15 (9.3)	49 (11.3)
Multiple races	8 (2.9)	3 (1.9)	11 (2.5)
Other race	2 (0.7)	1 (0.6)	3 (0.7)
White	166 (60.6)	118 (73.3)	284 (65.3)
Missing	3 (1.1)	1 (0.6)	4 (0.9)
Time between BHS/BFI completion and sample collection (days) (mean [SD])	40.08 (17.2)	39.63 (17.9)	39.91 (17.4)
Total BFI (mean [SD])	71.28 (17.0)	73.10 (17.0)	71.96 (17.0)
Missing (*n* [%])	8 (2.9)	4 (2.5)	12 (2.8)
Global BHS (mean [SD])	66.03 (20.1)	66.54 (20.5)	66.22 (20.3)
Missing (*n* [%])	0 (0.0)	1 (0.6)	1 (0.2)
Global BHS-ABA (mean [SD])	53.52 (25.3)	52.48 (25.7)	53.14 (25.4)
Missing (*n* [%])	7 (2.6)	7 (2.6)	14 (3.2)

Within the 274 participants with samples above the 80% KatharoSeq sequencing threshold, several participants were missing one or more bladder health scores (missing BHS: 0, missing BHS-ABA: 4, missing total BFI: 5, missing both BHS-ABA and total BFI: 3); however, scores for these measures were not significantly different between those above and below the threshold. Further descriptive characteristics are included in Table S1.

### Taxonomic distributions

**Fig 2:**
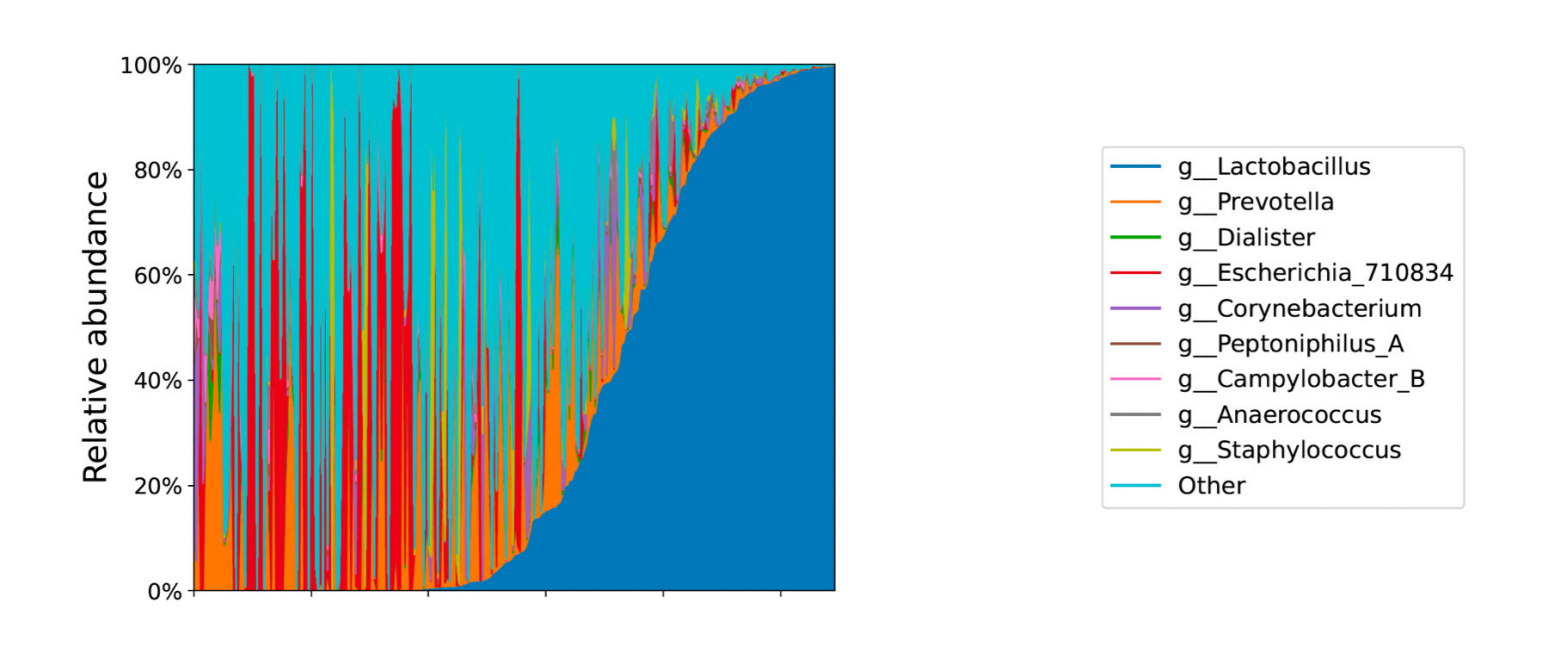
Taxonomic distribution of genera for self-collected urine samples for urobiome analysis in the RISE study ordered by *Lactobacillus* abundance. Legend shows the nine most abundant genera across individuals. Data are collapsed at the genus taxonomic level prior to plotting.

Figure 2 displays the taxonomic distribution of the genera for the 274 samples retained for analysis. These samples contained a diverse microbial distribution that included predominance by the genus *Lactobacillus*, with the majority of samples containing various proportions of mixed microbes.

### Distribution of bladder health scores relative to taxonomic distribution

**Fig 3:**
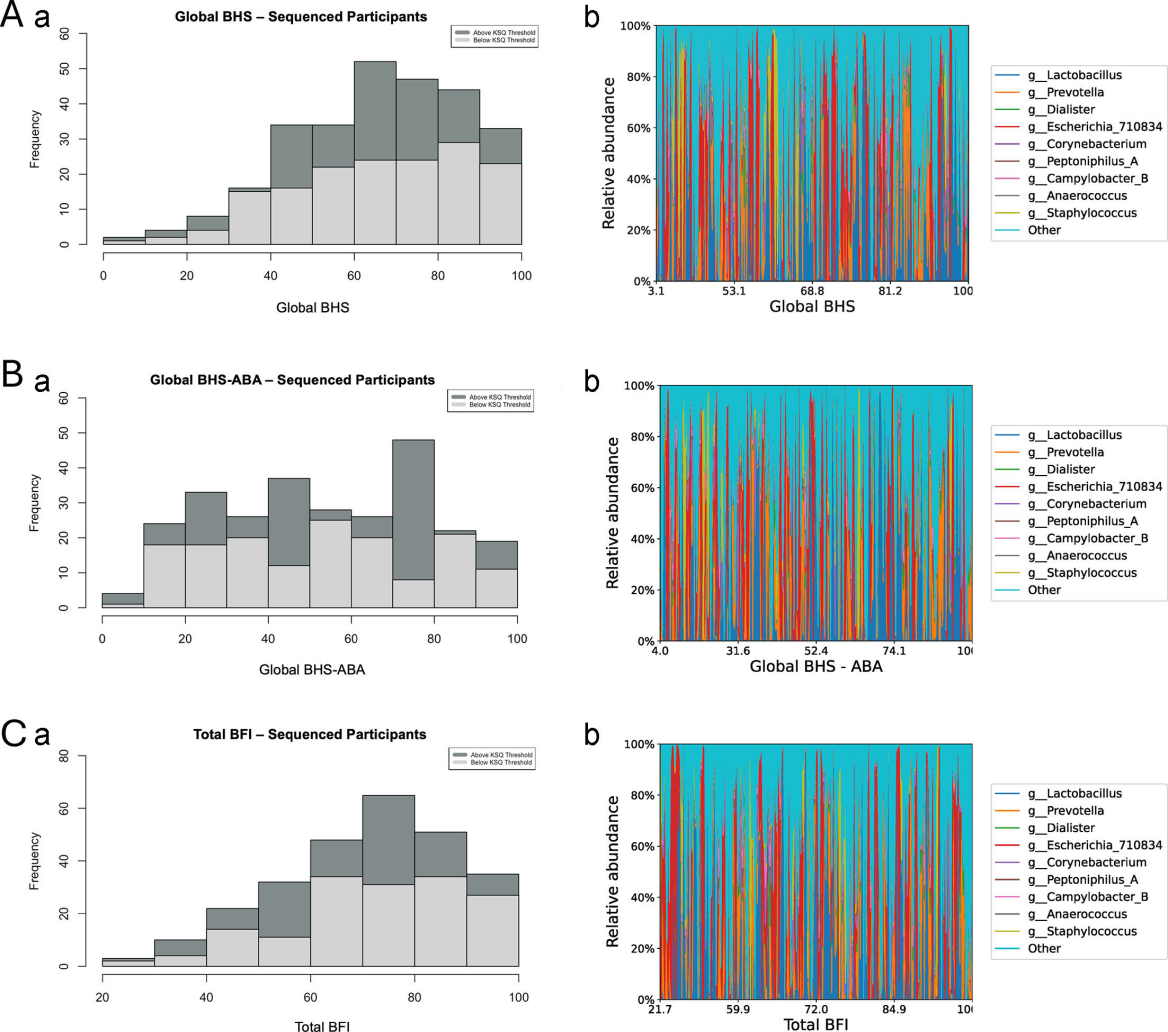
(**A**) (a) Global BHS for participants above and below the KatharoSeq threshold. (b) Nine most abundant genera vs global BHS scores in participant urine samples above the KatharoSeq threshold. (**B**) (a) Global BHS-ABA for participants above and below the KatharoSeq threshold. (b) Nine most abundant genera vs global BHS-ABA scores in participant urine samples above the KatharoSeq threshold. (**C**) (a) Total BFI for participants above and below the KatharoSeq threshold. (b) Nine most abundant genera vs total BFI scores in participant urine samples above the KatharoSeq threshold.

Figure 3 displays the distribution of bladder health scores separately for those samples above vs below KatharoSeq threshold and highlights the polymicrobial distribution across the bladder health spectrum. We did not detect any specific pattern of bacteria signatures that were clearly mapped to higher or lower bladder health scores.

### Associations of specific microbes with bladder health scores

**Fig 4:**
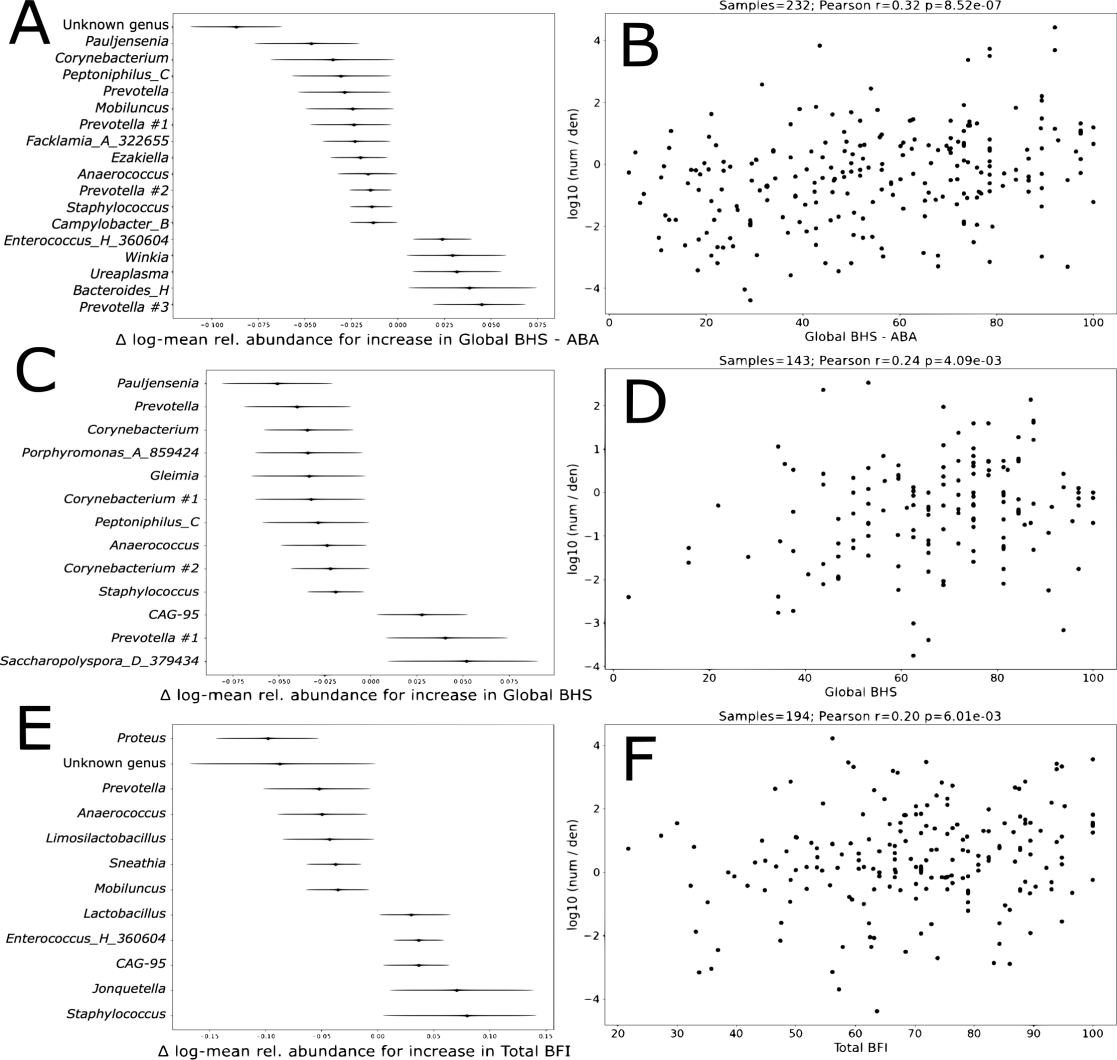
Microbes observed as differential with respect to the bladder health scores. (**A**) Taxon inference of microbes identified using BIRDMAn against global BHS-ABA; a taxon with a negative value has lower abundance associated with higher scores, whereas positive values indicate higher BHS scores were found to associate with higher abundance. (**B**) Correlation of global BHS-ABA with the log ratio of relative abundance for the taxa in panel **A**; Pearson’s *r* = 0.32, *P* = 8.52e − 7, *n* = 267. The sum of abundance of taxa whose intervals in panel **A** are positive forms the numerator of the ratio, whereas the sum of abundance of taxa whose intervals are negative forms the denominator. Sample size is dependent on samples with a nonzero numerator and denominator. (**C** and **D**) Like panels **A** and **B** but with global BHS; Pearson’s *r* = 0.24, *P* = 4.09e − 3, *n* = 274. (**E** and **F**) Like panels **A** and **B** but with overall BFI; Pearson’s r = 0.2, *P* = 6.01e − 3, n = 266.

Microbes observed as differential with respect to the bladder health scores, detected using BIRDMAn, are shown in Fig. 4 (11). Unit differences in measurement scores were tested against fold changes of abundance of each taxa using a negative binomial model. Taxa whose 95% credible intervals were both positive or negative for association with a measurement were retained. A taxon with a positive value indicates that higher measurement scores were found to be associated with larger taxon abundance, whereas a taxon with a negative value indicates that higher BHS scores are associated with lower taxon abundance (Fig. 4A, C, and E for global BHS-ABA, global BHS, and overall BFI, respectively). The composite microbe abundance log ratio for each bladder health measurement was then correlated with the bladder health measurement (Fig. 4B, D and E for global BHS-ABA, global BHS, and overall BFI, respectively: global BHS-ABA (*r* = 0.32, *P* < 0.001), global BHS (*r* = 0.24, *P* = 0.004), and total BFI (*r* = 0.20, *P* = 0.006).

*Pauljensemnia, Corynebacterium, Prevotella, Peptoniphillus, Anaerococcus*, and *Staphylococcus* were common genera associated with both lower global BHS and lower global BHS-ABA scores. We did not detect common bacteria that were associated with higher global BHS and higher global BHS-ABA scores.

*Staphylococcus, Jonquetella,* CAG-95*, Enterococcus* H*,* and *Lactobacillus* were significantly present in participants with higher total BFI scores, while *Proteus, Prevotella, Anaerococcus, Limosilactobacillus, Sneathia,* and *Mobiluncus* were associated with lower total BFI scores. Common bacteria associated with lower scores across all three measures of bladder health included *Prevotella* and *Anaerococcus. G*, indicating a genus that did not result in further identification at this level, was commonly seen in women with lower global BHS-ABA and total BFI scores. *Staphylococcus* was present in participants with higher total BFI scores yet lower global BHS and global BHS-ABA scores.

## DISCUSSION

This analysis advances urobiome research and suggests that the adult female urogenital microbiome is associated with aspects of bladder health. While there has been significant research and insight into the link between the urobiome and bladder disease, this robust analysis is the first to suggest a link between the female urogenital microbiome and the spectrum of bladder health. Bladder health, as assessed using validated measures, appears to be associated with several specific microbes. Two bacterial genera, *Prevotella* and *Anerococcus,* were associated with lower bladder health and function scores. While some bacterial genera were associated with higher bladder health and function scores, we did not identify any that were associated with all three measures of bladder health and function. In this study, we did not detect any specific patterns of bacteria signatures that were clearly mapped to higher or lower bladder health scores.

Not surprisingly, while the majority of samples derived from healthy community-dwelling women contained various proportions of mixed microbes, we did identify a predominance of *Lactobacillus*. This finding is consistent with other investigations of the urinary microbiome in healthy women (12). Previous studies demonstrate lower urinary microbial communities of *Lactobacillus* are associated with the presence of mixed urinary incontinence (13, 14) and interstitial cystitis/painful bladder syndrome (15, 16). The abundance of *Lactobacillus* in healthy women and lack of prominence in women with bladder conditions highlights the potential role this microbe plays in dysbiosis.

Our findings indicate that there is likely an influence of multiple community compositions on full-spectrum bladder health. It is important to note that our voided urine specimens represent the female urogenital microbiome, which could include microbes derived from the vulva and vagina and should be interpreted differently from other urinary specimens that better represent the bladder biome. This cross-contamination might contribute to the presence of *Staphylococcus* in women with higher bladder function scores and lower bladder health scores.

The challenges of large-scale sampling and sequencing of the urobiome have limited our understanding of the urobiome in healthy states. We previously reported our methods for overcoming these challenges as well as the feasibility of assessing the urobiome in a large sample of US women (9). The self-collection, shipping, sequencing, and analysis serve as a strong framework alongside our validated measures of bladder health. With rigorous methods and validated measures of bladder health, population-level urobiome research can be conducted. As this is one of the first studies to comprehensively assess the link between the urobiome and bladder health, it will provide a schematic for investigators to proceed with larger population-based studies.

This analysis benefits from multiple strengths, including a diverse study population with racial, ethnic, age, and geographic variability. Participants were characterized by a newly validated instrument. In addition, sample contributions from multiple study sites help expand the generalizability of the findings. Samples were acquired using a detailed, published protocol. Molecular preparation of the specimens was performed using a protocol designed specifically for low biomass samples, originally validated in the clean room of NASA’s Spacecraft Assembly Facility at the Jet Propulsion Lab (17), which allows for defining a limit of detection to distinguish true microbial signal from low-level common lab contaminants. While only 63% of our samples met the 80% Katharoseq threshold, it is reassuring that the distribution of unadjusted global bladder health scores was similar between participants with specimens above and below the prespecified threshold.

This analysis additionally used a new Bayesian differential abundance technique designed specifically for the properties of microbiome data. The sequencing protocols in this study, and most other microbiome studies, produce relative abundance data (more generally referred to as compositional data). These types of data are not compatible with classic statistical tests such as the *t*-test, because the relative abundance of a given microbe is dependent on all other microbes in the sample—the observations are not independent. The application of statistical methods that do not account for these properties increases both type 1 (false positive) and false negative (type 2) errors. Testing for all possible combinations of taxa for the numerator and the denominator results in an excessive number of combinations (hypotheses) to test. For example, all combinations of 4 taxa out of 100 for the numerator and denominator separately result in approximately 1.5e13 hypotheses to test. BIRDMAn is a principled method of using Bayesian statistics toward a set of taxa with evidence of association. Evaluation of those taxa, after the fact, using a log ratio and testing for a significant relationship to the variable of interest, is an approach not previously used in urobiome research (18).

While these findings suggest a relationship between bladder health and microbes, additional experiments in large diverse urobiome data sets are needed to refine the models and set of microbes which may associate with health and lifestyle, as has been imperative for the study of the human gut, skin, vaginal, and oral microbiomes (19–21). In particular, the collection of longitudinal data is critical to infer causality, better understand temporal relationships, and differentiate whether microbes are differential because a physiological change is imparting pressure on resident microbes, or if the microbes exhibit a change preceding a change in health. In addition, our findings do not specifically reflect the bladder microbiome, as voided specimens are known to include microbes from the bladder, urethra, and external genitalia.

Given the selection bias associated with only including participants who were willing and able to attend an in-person visit, these findings may not be generalizable to the full female population of community-dwelling women. Pragmatic limitations of the PLUS Research Consortium and the conduct of the study during the coronavirus disease 2019 pandemic limited the sample size. However, given the findings of the other RISE urogenital urobiome sample analyses that demonstrated the feasibility of self-collected, shipped samples and the concordance of those samples with self-collected samples collected in a research center setting, future studies can rigorously incorporate urinary biospecimen collection for larger population studies (9, 10). Second, this analysis evaluates the association of bladder health and the urogenital urobiome at a single point in time. Longitudinal assessments of RISE participants did not include additional biospecimen collection for financial and logistic reasons. Third, samples were not collected from individuals with active urinary tract infection or other known dysbiotic clinical states, although a proportion of the study participants reported various levels of lower urinary tract symptoms. However, this study was not designed to advance further understanding of the relationship of the urobiome in the presence of lower urinary tract symptoms. Focused research is warranted to replicate and advance these findings, in order to understand the implications for optimization of bladder health.

In conclusion, our data suggest that while we did not identify a “unique signature” specifically associated with high bladder health and function scores, there are significant associations between bacterial genera and low bladder health and function. Further investigation is warranted to investigate the role of urogenital microbes in promoting bladder health and preventing dysfunction.

## MATERIALS AND METHODS

### Methods of main RISE study and biospecimen substudy

The methods of the IRB-approved (UMN#00012315), observational RISE study and biospecimen substudy have been previously reported (8–10, 22). Briefly, eligible individuals were 18 years or older and born or identified as female, from the civilian, noninstitutionalized population residing in the 50 counties including or surrounding nine PLUS recruitment sites. Using population-based estimates drawn from a large marketing database (Acuity), potential participants were recruited from August 2022 to September 2023, using personalized mailings in both English and Spanish. After enrollment and completion of the two-part baseline research survey, which included the validated BHS and BFI instruments, participants were invited to attend an in-person evaluation that included self-collected voided urine (urogenital) samples. There were no exclusions for sample collection except that women were asked to avoid specimen collection during menstruation.

Participants self-reported baseline characteristics including demographics, medical history, medications, and potential risk or protective factors associated with bladder health. Bladder health and function were evaluated using the BHS and BFI (7).

### Urine specimen collection, shipping, and storage

In-person participants used a Peezy urine collection device to provide self-collected voided urine sample during research center visits that occurred between 14 June 2022 and 29 August 2023. The urine was transferred to a vial containing Assay Assure preservative, and samples were shipped at ambient temperature from PLUS research centers to a central laboratory (Rob Knight PhD at the University of California San Diego) via 2-Day FedEx. The shipping protocols, feasibility, and reliability of sample self-collection from this cohort have been reported (9, 10). Upon receipt, the lab examined the urine specimen tube to determine whether the specimen appeared visually viable. Samples were held at 4°C until transfer to the central laboratory where they were stored at −20°C until subsequent DNA extraction and sequencing.

### Urine sample DNA extraction and sequencing

#### Plating and gDNA extraction

Urine samples were placed in 4°C to thaw overnight before processing. Thawed urine samples were vortexed for 5 seconds to ensure sample homogenization, and 1 mL of urine was transferred into clean 1.0 mL Matrix Tubes (Thermo Fisher Scientific, Waltham, MA, USA) using a P1000 pipette. Matrix Tubes containing the urine samples were centrifuged for 10 min at 1,300 rcf at room temperature to form a pellet. After centrifugation, 800 µL of the supernatant was removed and discarded.

gDNA was extracted from the remaining sample using an extraction protocol optimized for Matrix Tubes. Briefly, 30 µL of zirconia-silica beads and 600 µL of MagMax Microbiome Ultra Lysis Buffer (Thermo Fisher Scientific, Waltham, MA, USA) were added to the Matrix Tubes containing the urine sample. The tubes were bead-beaten via a SpexMiniG (SPEX SamplePrep LLC, Metuchen, NJ, USA) for 2 min at 1,200 rpm, and then gDNA extraction proceeded using the MagMAX Microbiome Ultra Nucleic Acid Isolation Kit (Thermo Fisher Scientific, Waltham, MA, USA) on the KingFisher Flex instrument (Thermo Fisher Scientific, Waltham, MA, USA), as previously described (23). Extraction blanks, containing no sample input, were utilized as negative controls. Serially diluted *Variovorax paradoxus* positive controls with known cell counts were plated and extracted in order to utilize the KatharoSeq pipeline, which establishes a limit of detection to help distinguish urine samples from background or kit contaminant sequences (17).

#### 16S rRNA amplification and sequencing

16S rRNA amplification was performed on extracted gDNA using unique 515f-806r Golay barcodes adhering to Earth Microbiome Project standard protocols (https://earthmicrobiome.org/protocols-and-standards/dna-extraction-protocol/) and miniaturized PCR volumes, as previously described (14). Post-PCR amplicon libraries were equal volume pooled, PCR cleaned via the QIAquick PCR Purification Kit, (QIAGEN, Hilden, Germany), and quantified via the Qubit dsDNA Quantification Assay Kit (Thermo Fisher Scientific, Waltham, MA, USA), before being sequenced on a MiSeq using a 300 cycle reagent kit (v2) (Illumina, San Diego, CA, USA) with 2 × 151 paired-end reads.

#### Post-sequencing processing

MiSeq BCLs were converted to FASTQ using bcl2fastq in a managed workflow within Qiita. Generated data for this study are publicly available in Qiita under study ID 15405 (https://qiita.ucsd.edu/public/?study_id=15405) (24). Sequencing data have been deposited at EBI/ENA and are publicly available under accession number ERP160469 (https://www.ebi.ac.uk/ena/browser/view/PRJEB75918). The default amplicon data processing workflow was performed. Briefly, FASTQ files were demultiplexed and quality controlled using QIIME 1.9.1 (25), trimmed to 150 nucleotides, and denoised using Deblur version 2021.09 (26). Outside of Qiita, the Deblur amplicon sequence variants were mapped to the Greengenes2 2022.10 (27).

### KatharoSeq threshold

The KatharoSeq protocol was utilized using the QIIME2 plugin (17). An 80% KatharoSeq threshold was applied to the samples; samples that generated read counts below the 80% threshold were used for microbiome analyses. The minimum threshold of sequences per sample for Qiita preparation #16519 was 1581, and 5076 for Qiita preparation #16524.

### Analytic approaches—participant characteristics

Descriptive summaries were tabulated for participants with a sequenced sample and separately by those above vs below the pre-specified 80% KatharoSeq threshold. These included mean and standard deviation for continuous variables and frequency with percentage for categorical variables.

### Analytic approaches—microbiome

Community-wide measures of beta diversity can be sensitive to background noise if the association under test is subtle. Given the relatively high intra- and inter-subject variation in the urobiome, the analytic goal was to use a differential abundance method to identify a combination of taxa associated with three validated measures of bladder health.

The previously generated KatharoSeq-filtered feature tables were merged using QIIME 2 2023.9 (28) and the q2-feature-table’s merge action. All QIIME 2 actions described herein relied on the QIIME 2 version. Following the merge, samples that had been sequenced multiple times were collapsed using BIOM version 2.1.14 (29). The feature table was then filtered against Greengenes2 2022.10 (27) using the q2-greengenes2 filter-features action, in order to remove any amplicon sequence variants (ASVs) not represented in the phylogeny.

This table was then evaluated with BIRDMAn, a new compositionally aware, Bayesian differential abundance algorithm designed specifically for microbiome data (11). Prior to BIRDMAn, the feature table was filtered to the set of samples with non-null values for the bladder health measurement being examined, and any feature that was not present in at least 10% of the samples was omitted. The BIRDMAn technique evaluates each individual amplicon sequence variant under a Bayesian negative binomial model with log-link using a Markov chain Monte Carlo approach to sample from the posterior distribution. This technique is able to identify particular ASVs, which are associated with a bladder health measurement as a predictor against the background noise.

We identified top features as those amplicon sequence variants whose credible intervals are either both positive or both negative for each bladder health measurement. For each sample, we then created a ratio comprised of a composite (sum) of relative abundance of positively associated top feature variants (numerator) and composite of relative abundance of negatively associated top feature variants (denominator). The Pearson correlation of the log of this composite ratio with the bladder health measurement used to identify the top features was then evaluated.

## Data Availability

Sequence data with accompanying anonymized metadata for this study are publicly available in Qiita under study ID 15405 (https://qiita.ucsd.edu/public/?study_id=15405). Sequencing data have been deposited at EBI/ENA and are publicly available under accession number ERP160469 (https://www.ebi.ac.uk/ena/browser/view/PRJEB75918). Due to reporting requirements, investigators may note some redundancy in the metadata due to EBI submission requirements for stringent column naming and entry format. The original RISE data set is included for further research.

## SUPPLEMENTAL MATERIAL

Table S1 msystems.00558-25-s0001.docx Additional descriptive summary of 274 RISE Urobiome participants with a sequenced self-collected urine sample.

ASM does not own the copyrights to Supplemental Material that may be linked to, or accessed through, an article. The authors have granted ASM a non-exclusive, world-wide license to publish the Supplemental Material files. Please contact the corresponding author directly for reuse.
