# Supplementary material for: Bladder health and the urogenital microbiome in community-dwelling adult females
Source: mSystems. 2025 Oct 16;10(11):e00558-25. doi: 10.1128/msystems.00558-25 (PMC12625691; doi:10.1128/msystems.00558-25)
Supplement: Table S1 — Additional descriptive summary of 274 RISE Urobiome participants with a sequenced self-collected urine sample. [file msystems.00558-25-s0001.docx]

Supplement Table 1: Additional descriptive summary of 274 RISE Urobiome participants with a sequenced self-collected urine sample.

|  | **Above KatharoSeq Threshold** | **Not Above KatharoSeq Threshold** | **Sequenced** |
| --- | --- | --- | --- |
| n | 274 | 161 | 435 |
| Employment (%) |  |  |  |
| Full-time employed | 137 (50.0) | 78 (48.4) | 215 (49.4) |
| Part-time employed | 43 (15.7) | 31 (19.3) | 74 (17.0) |
| Homemaker | 8 (2.9) | 5 (3.1) | 13 (3.0) |
| Unemployed looking for work | 1 (0.4) | 0 (0.0) | 1 (0.2) |
| Unemployed not looking for work | 9 (3.3) | 3 (1.9) | 12 (2.8) |
| Student | 8 (2.9) | 5 (3.1) | 13 (3.0) |
| Retired | 59 (21.5) | 34 (21.1) | 93 (21.4) |
| Unable to work | 5 (1.8) | 1 (0.6) | 6 (1.4) |
| Missing | 4 (1.5) | 4 (2.5) | 8 (1.8) |
| Have Insurance (%) | 263 (96.0) | 155 (96.3) | 418 (96.1) |
| Insurance Type (%) |  |  |  |
| No insurance | 11 (4.0) | 6 (3.7) | 17 (3.9) |
| Medicaid only | 18 (6.6) | 4 (2.5) | 22 (5.1) |
| Medicare (no supplement) | 21 (7.7) | 7 (4.3) | 28 (6.4) |
| Medicare (with supplemental insurance) | 27 (9.9) | 23 (14.3) | 50 (11.5) |
| Medicare and Medicaid | 4 (1.5) | 0 (0.0) | 4 (0.9) |
| Tricare military VA or IHS | 3 (1.1) | 3 (1.9) | 6 (1.4) |
| Private | 180 (65.7) | 117 (72.7) | 297 (68.3) |
| Other | 10 (3.6) | 1 (0.6) | 11 (2.5) |
| Highest level of schooling completed (%) |  |  |  |
| No high school degree | 2 (0.7) | 0 (0.0) | 2 (0.5) |
| High school graduate or GED | 18 (6.6) | 7 (4.3) | 25 (5.7) |
| Some college or vocational training | 38 (13.9) | 21 (13.0) | 59 (13.6) |
| Vocational degree | 8 (2.9) | 1 (0.6) | 9 (2.1) |
| Associate's degree | 27 (9.9) | 14 (8.7) | 41 (9.4) |
| Bachelor's degree | 102 (37.2) | 55 (34.2) | 157 (36.1) |
| Graduate degree | 79 (28.8) | 62 (38.5) | 141 (32.4) |
| Missing | 0 (0.0) | 1 (0.6) | 1 (0.2) |
| Income (%) |  |  |  |
| Less than $10, 000 | 8 (2.9) | 3 (1.9) | 11 (2.5) |
| $10,000 to $14,999 | 7 (2.6) | 0 (0.0) | 7 (1.6) |
| $15,000 to $19,999 | 5 (1.8) | 1 (0.6) | 6 (1.4) |
| $20,000 to $24,999 | 9 (3.3) | 3 (1.9) | 12 (2.8) |
| $25,000 to $29,999 | 10 (3.6) | 2 (1.2) | 12 (2.8) |
| $30,000 to $34,999 | 11 (4.0) | 4 (2.5) | 15 (3.4) |
| $35,000 to $39,999 | 12 (4.4) | 1 (0.6) | 13 (3.0) |
| $40,000 to $44,999 | 10 (3.6) | 3 (1.9) | 13 (3.0) |
| $45,000 to $49,999 | 3 (1.1) | 2 (1.2) | 5 (1.1) |
| $50,000 to $74,999 | 37 (13.5) | 22 (13.7) | 59 (13.6) |
| $75,000 to $99,999 | 43 (15.7) | 26 (16.1) | 69 (15.9) |
| $100,000 to $149,999 | 48 (17.5) | 22 (13.7) | 70 (16.1) |
| $150,000 or more | 41 (15.0) | 46 (28.6) | 87 (20.0) |
| Don't know | 15 (5.5) | 15 (9.3) | 30 (6.9) |
| Missing | 15 (5.5) | 11 (6.8) | 26 (6.0) |
| Meets 100% of Federal Poverty Level (%) |  |  |  |
| 0-100 | 20 (7.3) | 6 (3.7) | 26 (6.0) |
| 100+ | 222 (81.0) | 128 (79.5) | 350 (80.5) |
| Missing | 32 (11.7) | 27 (16.8) | 59 (13.6) |
| Meets 300% of FPL (%) |  |  |  |
| 0-300 | 93 (33.9) | 28 (17.4) | 121 (27.8) |
| 300+ | 149 (54.4) | 106 (65.8) | 255 (58.6) |
| Missing | 32 (11.7) | 27 (16.8) | 59 (13.6) |
| Perceived Financial Stability (%) |  |  |  |
| Not have enough money to make ends meet | 26 (9.5) | 6 (3.7) | 32 (7.4) |
| Have just enough money to make ends meet | 77 (28.1) | 34 (21.1) | 111 (25.5) |
| Have some money left over | 100 (36.5) | 58 (36.0) | 158 (36.3) |
| Have more than enough money left over | 63 (23.0) | 55 (34.2) | 118 (27.1) |
| Missing | 8 (2.9) | 8 (5.0) | 16 (3.7) |

Supplemental Table 2 - The STORMS checklist. An editable version for adaptation and inclusion in publications is available from <https://stormsmicrobiome.org>
